# Microbiota and bile acid profiles in retinoic acid-primed mice that exhibit accelerated liver regeneration

**DOI:** 10.18632/oncotarget.6665

**Published:** 2015-12-18

**Authors:** Hui-Xin Liu, Ying Hu, Yu-Jui Yvonne Wan

**Affiliations:** ^1^ Department of Medical Pathology and Laboratory Medicine, University of California, Davis, Sacramento, CA, USA

**Keywords:** partial hepatectomy, lipid homeostasis, energy metabolism, gut-liver axis, fibroblast growth factor 21, Pathology Section

## Abstract

**Background & Aims:**

All-trans Retinoic acid (RA) regulates hepatic lipid and bile acid homeostasis. Similar to bile acid (BA), RA accelerates partial hepatectomy (PHx)-induced liver regeneration. Because there is a bidirectional regulatory relationship between gut microbiota and BA synthesis, we examined the effect of RA in altering the gut microbial population and BA composition and established their relationship with hepatic biological processes during the active phases of liver regeneration.

**Methods:**

C57BL/6 mice were treated with RA orally followed by 2/3 PHx. The roles of RA in shifting gut microbiota and BA profiles as well as hepatocyte metabolism and proliferation were studied.

**Results:**

RA-primed mice exhibited accelerated hepatocyte proliferation revealed by higher numbers of Ki67-positive cells compared to untreated mice. Firmicutes and Bacteroidetes phyla dominated the gut microbial community (>85%) in both control and RA-primed mice after PHx. RA reduced the ratio of Firmicutes to Bacteroidetes, which was associated with a lean phenotype. Consistently, RA-primed mice lacked transient lipid accumulation normally found in regenerating livers. In addition, RA altered BA homeostasis and shifted BA profiles by increasing the ratio of hydrophilic to hydrophobic BAs in regenerating livers. Accordingly, metabolic regulators fibroblast growth factor 21, Sirtuin1, and their downstream targets AMPK and ERK1/2 were more robustly activated in RA-primed than unprimed regenerating livers.

**Conclusions:**

Priming mice with RA resulted in a lean microbiota composition and hydrophilic BA profiles, which were associated with facilitated metabolism and enhanced cell proliferation.

## INTRODUCTION

The gut microbiota plays a crucial role in promoting cell proliferation. For examples, germ-free mice have reduced intestinal epithelial cell turnover due to decreased proliferation, apoptosis, and crypt-to-tip cellular migration [1]. Gut microbiota-generated toll-like receptor signals are required for injured tissues to regenerate and regain homeostasis [2]. Moreover, conventional mice have reduced cancer incidence compared with germ-free mice [3], and an increased bacterial load is found in colonic biopsies from patients with colorectal adenoma or cancer [4]. One mechanism by which bacteria promote cell proliferation is through the stimulatory effect of gram negative bacteria-generated lipopolysaccharide (LPS) on DNA synthesis [5]. It has been shown in mice that hepatic DNA synthesis is impaired when gut-derived LPS is blocked from reaching the liver [6]. Furthermore, both germ-free and LPS-resistant mice exhibit delayed liver regeneration after liver resection, which can be rescued by LPS administration [7]. In addition to proliferation, the gut microbiota modulates host metabolic phenotype and participates in microbial-host co-metabolism [8]. Alterations in gut bacterial communities are associated with metabolic disorders [9], metabolic syndrome [10], obesity [11-13], and nonalcoholic steatohepatitis [14]. Taken together, there is a close relationship between microbial-host metabolism and tissue injury as well as regeneration [15, 16].

Besides LPS, another gut-derived signaling that contributes to liver regeneration is bile acid (BA). There is substantial metabolic demand during liver regeneration, and proper function of intestinal nutrient absorption-mediated by BAs is essential for normal liver repair. The hydrophobic nature of BAs facilitates lipid absorption while conjugated hydrophilic BAs are more effective in lipid emulsification [17]. Fine-tuned lipid and BA homeostasis is important in supporting liver regeneration. Steatosis dampens liver repair capability [18, 19], and leptin-inhibited hepatic fat accumulation also impairs partial hepatectomy (PHx)-induced liver regeneration [20]. Additionally, BA overload induces liver injury in regenerating liver and increases mortality [21-23]. However, BA depletion by cholestyramine reduces liver regeneration capability, and the restored liver mass is reduced by 50% [24]. Together, maintaining BA homeostasis is crucial for normal progression of liver regeneration [15, 16].

Hepatic enzymes together with bacteria enzymes are responsible for generating various kinds of BAs. Microbiota plays a pivotal role in BA synthesis by increasing BA composition diversity *via* de-conjugation, dehydrogenation, dihydroxylation, and sulfation of primary BAs in the gastrointestinal (GI) tract [25]. Bile salt hydrolase (BSH) detected from *Bacteroides*, *Bifidobacterium*, and *Lactobacillus* catalyzes the de-conjugation of BAs to liberate free primary BAs. Furthermore, several bacteria belonging to the Firmicutes phylum have hydroxysteroid dehydrogenase, which mediates BA oxidation and epimerization. The compositions of BAs between germ-free and conventional rats are markedly different [26]. A cross-sectional study of patients with cirrhosis showed elevated primary BAs and *Enterobacteriaceae* and diminished 7α-dehydroxylating bacteria including *Lachonospiraceae, Ruminococcaceae,* and *Blautia,* which convert free BAs to secondary BAs [27]. Gut microbiota-generated deoxycholic acid (DCA) and lithocholic acid (LCA) induce cell proliferation and GI cancers [28]. These findings clearly indicate that gut microbiota modulate host BA synthesis and diversity, which in turn influence liver function.

Similar to BAs, all-trans retinoic acid (RA), naturally presented in the GI tract, has a profound effect in regulating lipid homeostasis [29, 30]. We have previously shown that RA can facilitate PHx-induced liver regeneration *via* inducing cell cycle gene expression, which was comparable to the liver regeneration effect of cholic acid (CA) [24, 31]. Because of the intimate relationship between gut-derived signaling and liver regeneration, we hypothesize that RA may regulate gut microbiota and BA composition thereby promoting hepatocyte proliferation in regenerating livers. To test this hypothesis, we analyzed the gut microbiota and BA composition to understand their potential role in PHx-induced liver regeneration in response to RA treatment. Our data showed that priming mice with RA resulted in a lean microbiota composition and hydrophilic BA profile, which were associated with facilitated metabolism and accelerated hepatocyte proliferation.

## RESULTS

### Priming mice with RA accelerated liver regeneration

**Figure 1:**
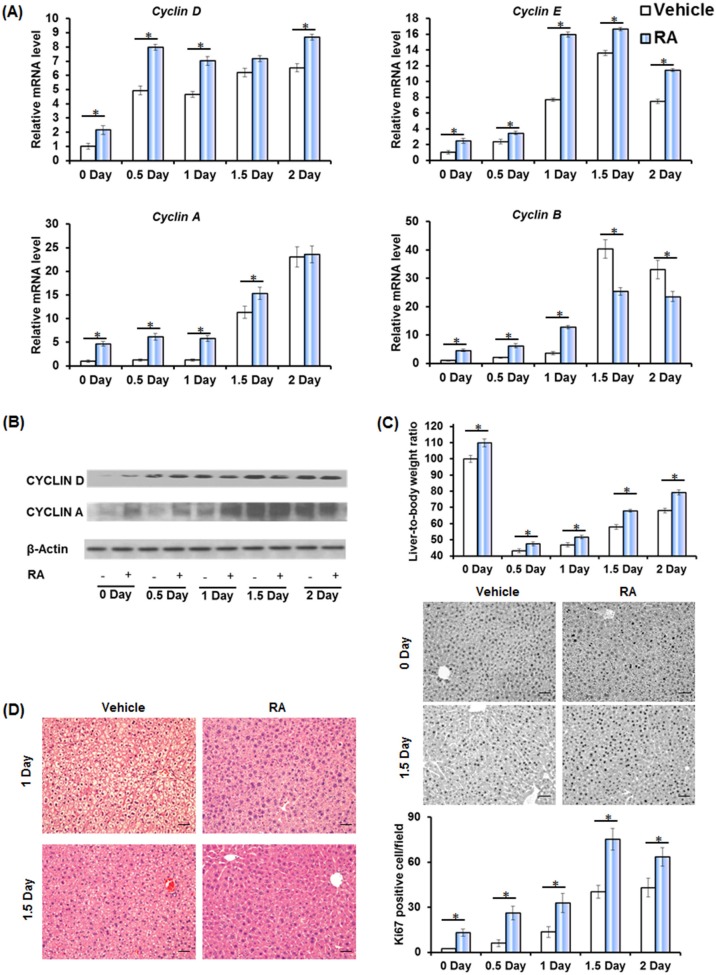
Accelerated liver regeneration in RA-primed mice Wild type (WT) mice were treated with all-trans retinoic acid (RA) or vehicle by oral gavage 48 hours prior to partial hepatectomy (*n* = 4). **A.** Quantitative RT-PCR analyses of hepatic mRNA level of *Cyclin D*, *Cyclin E*, *Cyclin A*, *Cyclin B*. **B.** Immunoblotting of liver extracts using anti-CYCLIN D and CYCLIN A antibodies. **C.** Liver-to-body weight ratios and immunohistochemistry of liver sections for Ki67 staining (20×) are shown. The number of Ki67-labeled nuclei was counted in at least 10 low-magnification (20×) microscope fields for each liver section. **D.** H&E staining of liver sections. Representative images are shown (20×). Means ± SD are graphed with * indicating *p* < 0.05.

Wild type mice received an oral gavage of RA or vehicle, and PHx was performed 48 hours later. RA-primed mouse livers had higher induction of cell cycle gene expressions, including *Cyclin D* (0-1, 2 day), *Cyclin E* (0-2 day), *Cyclin A* (0-1.5 day), and *Cyclin B* (0-2 day) (Figure 1A). The hepatic protein levels of CYCLIN D (0-0.5 day) and CYCLIN A (0-1 day) also showed increased induction (Figure 1B). RA-induced cell cycle gene expression correlated with enhanced liver regrowth and proliferation, as shown by greater liver-to-body weight ratio and numbers of Ki67-positive cells at all studied times (Figure 1C). Together, RA administration accelerated liver regeneration. Interesting, lipid droplets, usually occurring 1-1.5 day after PHx [18], were absent in RA-primed mouse livers (Figure 1D), indicating a regulatory effect on lipid metabolism by RA during liver regeneration.

### Priming mice with RA altered gut microbiota during liver regeneration

**Figure 2:**
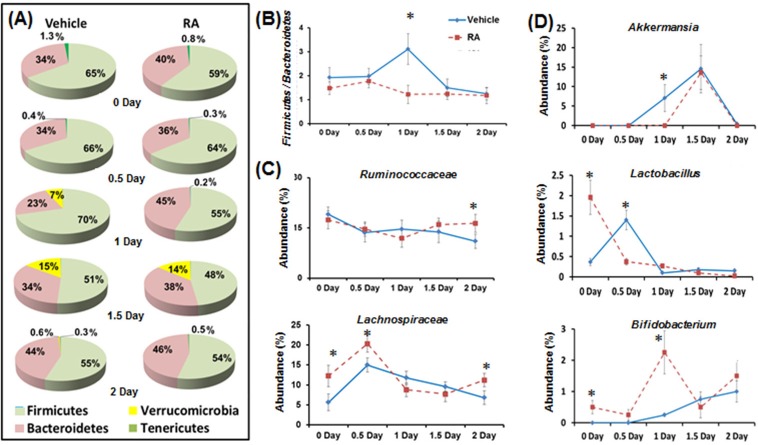
The effect of RA on gut microbiota during liver regeneration RA-primed mice showed altered gut microbiota composition **A.**, the ratio of Firmicutes to Bacteroidetes **B.**, family levels of *Ruminococcaceae* and *Lachnospiraceae* **C.**, and genus levels of *Akkemansia*, *Lactobacillus*, and *Bifidobacterium* **D.**. Means ± SD are graphed with * indicating *p* < 0.05.

Firmicutes and Bacteroidetes phyla dominated the microbial community (> 85%) in both control and RA-primed mice during liver regeneration (Figure 2A). In control mice, there was a transient increase in the abundance of *Verrucomicrobia* which rose to 7% on day 1, continued increasing to 15% on day 1.5, and contracted to 0.6% on day 2. However, in RA-primed mice, the *Verrucomicrobia* increase was only detected on 1.5 day after PHx (Figure 2A). RA-treated mice had a reduced ratio of Firmicutes to Bacteroidetes 1 day after surgery (2-fold increase in *Bacteroidetes*) (Figure 2B). The *Ruminococcaceae* and *Lachnospiraceae* are two of the most abundant families from the Firmicutes phyla found in the mammalian intestine, and are associated with intestinal health [32]. Higher levels of *Ruminococcaceae* (day 2) and *Lachnospiraceae* (0, 0.5, 2 days) were observed in RA-primed mice (Figure 2C). At the genus level, *Akkermansia* from the *Verrucomicrobia* phyla appeared on day 1 (7%), increased to 14.6% on day 1.5, and became undetectable on day 2 in the control mice. The appearance of *Akkermansia* was delayed in RA-treated mice (Figure 2D). RA increased the abundance of genus *Lactobacillus* by four folds on day zero (Figure 2D). The abundance of *Bifidobacterium* was also dramatically higher in RA-primed mice on day zero and 1 day after PHx (> 9 folds) (Figure 2D).

### RA regulates the expression of BA homeostasis genes during liver generation

**Figure 3:**
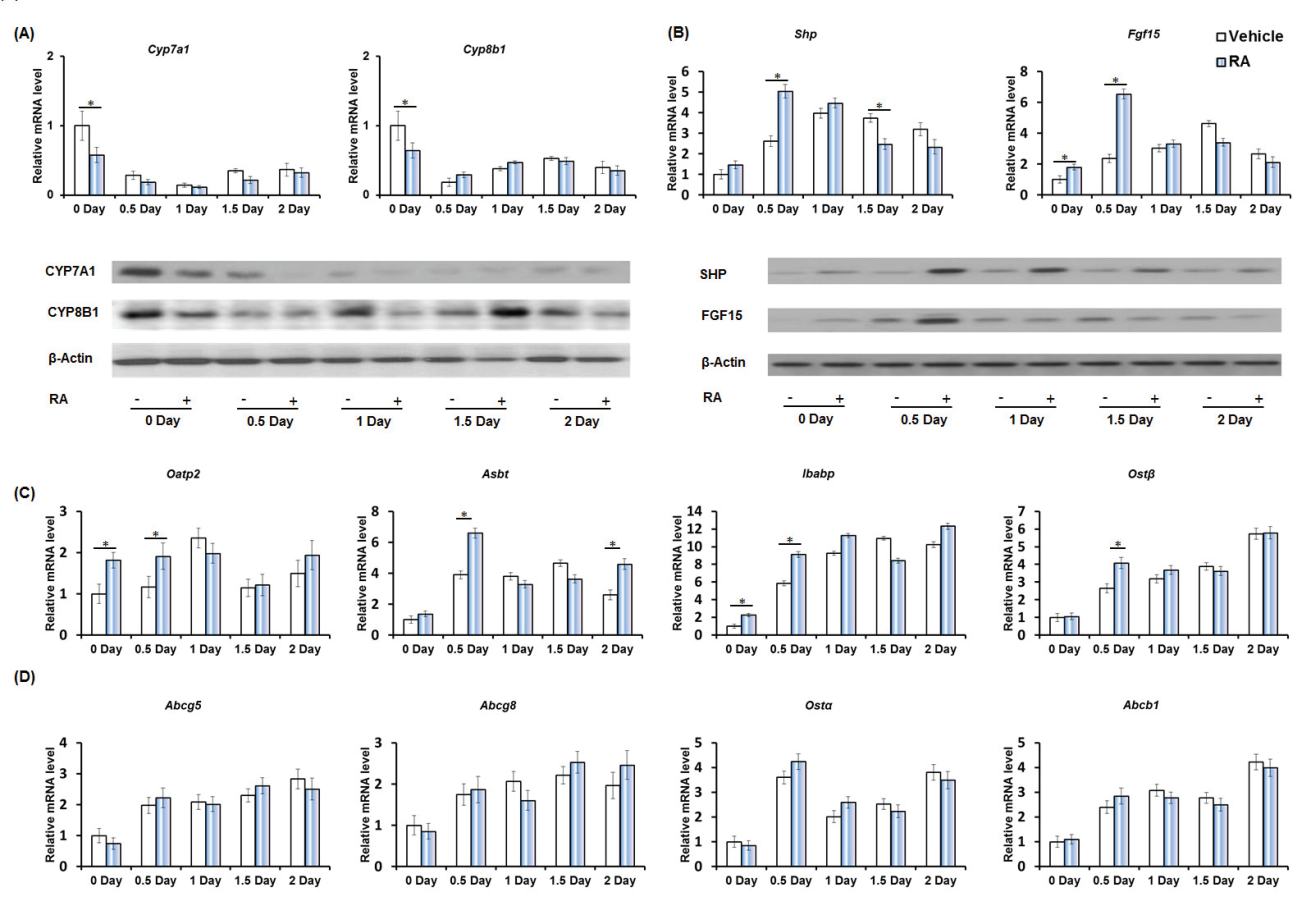
The effect of RA on the expression of genes regulating bile acid homeostasis during liver regeneration **A.** Quantitative RT-PCR analyses of hepatic mRNA abundance of BA synthesis genes *Cyp7a1* and *Cyp8b1*, and immunoblotting of liver extracts using CYP7A1 and CYP8B1 antibodies. **B.** Quantitative RT-PCR analyses of ileal mRNA level of BA regulator *Shp* and *Fgf15*, and immunoblotting of ileal extracts using anti-SHP and FGF15 antibodies. **C.** Quantitative RT-PCR analyses of ileal mRNA level of BA transporter *Oatp2*, *Abst*, *Ibabp*, and *Ostβ*. **D.** RA had no effects on ileal mRNA level of BA transporter *Abcg5*, *Abcg8*, *Ostα*, and *Abcb1*. Means ± SD are graphed with * indicating *p* < 0.05.

Studies of the hepatic BA synthesis pathway revealed that priming mice with RA reduced the expression of hepatic cholesterol 7α-hydroxylase (*Cyp7a1*) and sterol 12α-hydroxylase (*Cyp8b1*) on day zero. At the protein level, RA reduced CYP7A1 (0-1 day) and CYP8B1 (0, 1, 2 day), but increased CYP8B1 on day 1.5 (Figure 3A).

In the ileum, PHx itself induced small heterodimer partner (*Shp*) and fibroblast growth factor (*Fgf15*) expression immediately after surgery (day 0.5) and this induction was sustained throughout the active phase of hepatocyte proliferation. Priming mice with RA further increased the expression of *Shp* (0.5 day) and *Fgf15* (0-0.5 day) indicating an effect of RA in modulating BA-mediated FXR signaling in the ileum during liver regeneration (Figure 3B). SHP and FGF15 protein levels showed a consistent increase with the biggest difference on 0.5 day (Figure 3B). Similar patterns were also observed for the expression of ileal organic anion transporting polypeptide-2 (*Oatp2*), apical sodium-dependent bile acid transporter (*Asbt*), and ileal bile acid-binding protein (*Ibabp*). The expression of these transporters was induced after PHx, indicating increased intestinal BA uptake during liver regeneration [33]. Furthermore, RA enhanced the expression of *Oatp2* (0-0.5 day), *Asbt* (0.5, 2 day), and *Ibabp* (0-1 day) (Figure 3C). Organic solute transporter beta (*Ostβ*), a basolateral BA efflux transporter, was also induced in the ileum during liver regeneration, and its induction was further enhanced by RA-priming (day 0.5) (Figure 3C). RA had no effect in regulating ATP-binding cassette, sub-family g, member 5 (*Abcg5*), *Abcg8*, *Ostα*, and *Abca1 expression*. However, these genes were induced in regenerating livers (Figure 3D).

### RA alters hepatic BA profile during liver regeneration

**Figure 4:**
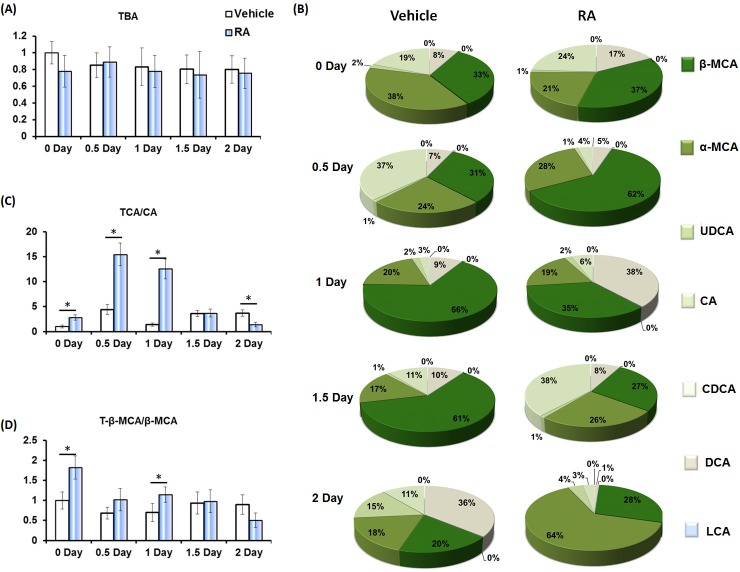
The effect of RA on hepatic bile acid composition during liver regeneration **A.** RA had no effect on hepatic total bile acids (TBA); **B.** RA shifted the percentages of indicated free bile acids in vehicle and RA-treated mice after PHx; **C.** the ratio of taurocholic acid (TCA) to cholic acid (CA), and **D.** the ratio of tauro-β-muricholic acid (T-β-MCA) to β-muricholic acid (β-MCA). Means ± SD are graphed with * indicating *p* < 0.05.

Hepatic BAs quantification showed that priming mice with RA had no effect on the amount of total bile acids (TBA) (Figure 4A). However, RA treatment shifted the composition of free BAs (Figure 4B). Tauro-conjugation of BAs increases their hydrophilic/hydrophobic ratio, solubility and lipid emulsification [17], and the taurocholic acid (TCA)/CA ratio was significantly higher in RA-treated mice compared to controls (Figure 4C). The tauro-β-muricholic acid (T-β-MCA) to β-muricholic acid (β-MCA) ratio, associated with increasing lipid emulsion efficiency [8, 17], was also higher in RA-primed mice compared to controls on day zero and 1 day after PHx (Figure 4D).

### RA regulates energy homeostasis by inducing FGF21 signaling

**Figure 5:**
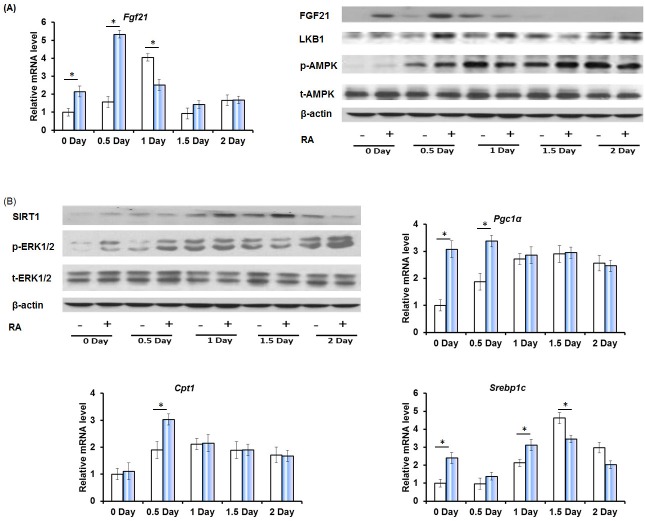
The effect of RA on the expression of factors that regulate hepatic metabolism and cell proliferation during liver regeneration **A.** Quantitative RT-PCR analyses of hepatic mRNA abundance of *Fgf21*, and immunoblotting of liver extracts using FGF21, LKB1, p-AMPK, and t-AMPK antibodies. **B.** Immunoblotting of liver extracts using SIRT1, p-ERK1/2, and t-ERK1/2 antibodies, and Quantitative RT-PCR analyses of hepatic mRNA abundance of *Pgc1α, Cpt1,* and *Srebp1c*. Means ± SD are graphed with * indicating *p* < 0.05.

Fibroblast growth factor 21 (FGF21) is a master metabolic regulator that has a striking ability to reverse obesity, diabetes, and injury as well as prolong life span [34]. FGF21 is transiently induced during the early phase of liver regeneration [23]. RA treatment resulted in early induction of FGF21 mRNA and protein levels after PHx (Figure 5A). Serine/threonine kinase 11 (LKB1), a downstream target of FGF21, also showed earlier induction in RA-primed mice after PHx. FGF21 increases the phosphorylation of AMP-activated protein kinase (AMPK) through LKB1 [35]. Higher phospho-AMPK level was found in RA-treated mice on day 0, 0.5, and 1.5, but RA did not alter total AMPK protein level (Figure 5A). AMPK has been shown to induce NAD^+^-dependent deacetylase sirtuin 1 (SIRT1) activity (day 1 and 2), resulting in modulation of downstream targets including peroxisome proliferator-activated receptor-γ coactivator 1α (*Pgc1α*), carnitine acyltransferase 1 (*Cpt1*), and sterol regulatory element-binding transcription factor 1c (*Srebp1c*) [35]. Increased FGF21 was accompanied by elevated SIRT1, *Pgc1α*, and *Cpt1* 0-1 day after PHx (Figure 5B). In addition, PHx did not affect the total extracellular-signal-regulated kinase 1 and 2 (ERK1/2) level, but rather induced ERK1/2 phosphorylation, which was further increased by RA on day 0 and 0.5 day (Figure 5B).

## DISCUSSION

Emerging evidence indicates that bidirectional communication exists between the gut microbiota and BAs through a “gut-liver axis” [1, 25]. The gut microbiome has increasingly been acknowledged as a novel contributor to host metabolism [25, 27]. Mounting evidence in mice and humans supports that the gut microbiota is linked with metabolic disorders such as diabetes and obesity [1, 27]. Liver resection changes lipid and BA metabolism during a period when increased energy production is needed to meet the metabolic demands of cell proliferation [36]. We demonstrated, for the first time, that RA altered the gut microbiota composition and BA profile, which was accompanied by an increase in energy metabolism, beneficial for liver regeneration.

Alterations in gut microbiota composition have been implicated in metabolic disorders, and specific bacterial phylotypes or metabolic activities may be beneficial or detrimental to patients with obesity [1, 8]. Firmicutes and Bacteroidetes are the two most dominant bacterial phyla that affect the efficiency of host energy extraction and are linked with excess adiposity in both mice and humans [13, 37]. Obese mice have more Firmicutes and fewer Bacteroidetes compared to lean mice. The role of RA in regulating lipid homoestasis had been defined by genomic binding and transcriptome profiling [29]. Decreased lipid accumulation and the ratio of Firmicutes to Bacteroidetes presented in RA-primed mice are associated with accelerated liver regeneration, indicating beneficial lipid metabolism induced by RA during liver regeneration. Phyla *Verrucomicrobia* is occasionally observed, and is capable of pathogenic activity with growth inhibition toward the host [38, 39]. *Lachnospiraceae* and *Ruminococcaceae* are the most abundant Firmicute families in the intestines, accounting for roughly 50% and 30% of phylotypes, respectively in healthy adults [40, 41]. *Lachnospiraceae* and *Ruminococcaceae*, associated with the production of butyrate necessary for colonic epithelial health, have been shown to be depleted in inflammatory bowel disease patients [42, 43]. The gut microbiota exerts a strong influence on energy homeostasis due to bacteria-produced butyrate which serves as an energy source for colonocytes [44]. The increased *Lachnospiraceae* and *Ruminococcaceae* due to RA treatment may increase butyrate production, which may benefit liver regeneration.

BAs are efficiently conserved under normal conditions by a process termed enterohepatic recirculation [33]. Conjugated and unconjugated BAs are reabsorbed by passive diffusion along the entire gut and by active transport in the terminal ileum. BA overload has been observed after liver resection, and *Cyp7a1* and *Cyp8b1* inhibition indicate the necessity to down-regulate BA synthesis during liver regeneration [23, 24]. *Cyp7a1* expression can be regulated by a negative feedback loop involving intestinal BA signaling [22, 33]. RA-mediated induction of SHP and *Fgf15* has been shown to mediate this suppression [30]. During liver regeneration, RA can boost this *Fxr*-mediated pathway to further inhibit hepatic BA synthesis. *Asbt* absorbs the majority of BAs available in the adjacent lumen of the ileum and is critical for intestinal BA reabsorption during enterohepatic recirculation [45]. Mutation or inhibition of *Asbt* causes BA malabsorption and interruption of the enterohepatic circulation of BAs resulting in chronic diarrhea, steatorrhea, and fat-soluble vitamin malabsorption [45, 46]. In the intestines, BAs are transported by *Ibabp,* after which they are secreted back into circulation by several transporters including *Ostα*/*β*. The up regulation of these genes found in regenerating livers can accelerate BA circulation, thereby protecting hepatocytes against high concentration of BAs. Together, the presented data illustrated that in response to liver resection, the enterohepatic recirculation adapted to the immediate BA overload by inhibiting hepatic BA synthesis and inducing intestinal BAs transporters, both of which were enhanced by RA.

It is possible that through gut microbiota-modulated BA composition, RA facilitated metabolism, which contributed to accelerated liver regeneration. Metabolism of BAs by gut microbes increases BA diversity, thus not only affecting BA and lipid homeostasis, but also other aspects of host health [8]. For example, BSH activity, produced in significant amounts by *Lactobacillus* and *Bifidobacterium,* could be a way for bacterial species to detoxify BAs [8]. In addition, the dehydroxylation of chenodeoxycholic acid (CDCA) forms LCA, which is toxic to hepatocytes and has been linked to colon carcinogenesis [28]. Conversely, epimerization of CDCA generates ursodeoxycholic acid (UDCA), which is chemoprotective and is used to treat cholesterol gallstone [47]. Furthermore, conjugated BAs reduce the hydrophobic/hydrophilic ratio and are more effective in lipid emulsification and micelle formation. The presented data revealed a shift towards hydrophilic and conjugated BAs in RA-treated mice. Together, RA treatment may exert beneficial BA metabolism by intestinal microbes for liver regeneration.

FGF21 is required for activation of hepatic lipid oxidation, triglyceride clearance, and ketogenesis during fasting [48]. Administration of FGF21 to rodents with diet-induced or genetic obesity and diabetes resulted in potent antihyperglycemic and triglyceride lowering effects [35]. Forced FGF21 expression in partial hepatectomized hPPARα^PAC^ mice reduced hepatic steatosis, prevented focal necrosis, and restored liver mass [23]. LKB1 and AMPK are required for FGF21-mediated energy regulation of mitochondrial oxidative function [35] *via Pgc1α, Cpt1,* and *Srebp1c* [49, 50]. Inhibition of AMPK activity attenuated FGF21-stimulated increase in *Cpt1* and *Pgc1α* gene expression [35]. Both AMPK and SIRT1 act together with *Pgc1α*, the master regulator of mitochondrial biogenesis, to modulate energy homeostasis in response to environmental and nutritional stimuli [35]. The sustained activation of ERK1/2, can also be regulated by FGF21 to induce stimulatory cell cycle regulators [51]. Transient accumulation of hepatocellular fat during the early stage of liver regeneration has been proposed to serve as an β-oxidation energy source for hepatocellular proliferation [52]. Consistently, infusion of octanoylcarnitine, an inhibitor of *β*-oxidation, decreased DNA synthesis and energy charge levels in regenerating livers [53]. Mice treated with RA seem to have increased efficiency in lipid transport and metabolism, which is suggested by a lack of lipid accumulation and accelerated hepatocyte proliferation.

In summary, our data showed that RA-accelerated liver regeneration was accompanied by a decreased ratio of Firmicutes to Bacteroidetes, which is known to be associated with a lean phenotype. Consistently, RA-primed mice lacked the transient lipid accumulation normally found in regenerating mouse livers. In addition, RA altered BA signaling, enhanced enterohepatic recirculation of BAs, and increased the hydrophilic/hydrophobic ratio of BAs in the regenerating livers, potentially yielding higher solubility and lipid emulsification capability in RA-primed mice. RA-priming afforded more efficient lipid circulation and induction of the FGF21-LKB1-AMPK pathway, which boosted the energy metabolism necessary for liver regeneration. A better mechanistic understanding of the proliferative effects exerted by RA through modulating gut microbiota composition may allow for the development of therapeutics to promote liver regeneration in the clinical setting.

## MATERIALS AND METHODS

### Animal

Male C57BL/6 mice (3-5 months old) were housed in steel microisolator cages at 22°C with a 12-hour light/dark cycle. Food and water were provided *ad libitum* throughout study. RA (25 μg/gram of body weight, one time) (Sigma-Aldrich Corp., St. Louis, MO) or vehicle control (carboxymethyl cellulose) (Sigma-Aldrich Corp., St. Louis, MO) was administered to the mice by gavage. Forty-eight hours after treatment, 2/3 PHx was performed [31, 54, 55]. Mice were killed 0.5, 1, 1.5, and 2 days after surgery (*n* = 4) covering the time when hepatocytes are actively proliferating. Zero time point mice, which were killed immediately after PHx, served as controls (0 day). All animal experiments were conducted in accordance with the National Institutes of Health Guide for the Care and Use of Laboratory Animals under protocols approved by the Institutional Animal Care and Use Committee of the University of California, Davis.

### Sequencing and analysis of gut microbial communities in the cecal contents

Cecal DNA was extracted using the MoBio power soil kit (MoBio) according to the manufacturer's protocol. Multiplexed DNA libraries were prepared according to a previously described protocol [56]. In brief, the V4 domain was amplified using primers 515F and 806R, a set of prokaryotic universal primers that was well-validated and used as the standard protocol for the Earth Microbiome Project (http://www.earthmicrobiome.org/), with a unique identifier sequence for each sample. Polymerase chain reaction (PCR) mixtures contained 1 Unit Kapa2G Robust Hot Start Polymerase (Kapa Biosystems), 1.5 mM MgCl_2_, 10 pmol of each primer, and 2 ng of DNA. PCR included 95°C for 2 minutes, followed by 30 cycles of 95°C for 15 seconds, 55°C for 30 seconds, and 72°C for 45 seconds and a final extension of 72°C for 3 minutes. The amplicon libraries were sequenced using 2 × 250-bp paired-end protocol on an Illumina Miseq system. Sequencing reads were trimmed of their barcodes, demultiplexed and combined using custom Perl scripts and aligned to the Greengenes (release 13_5) database using Qiime with default parameters.

### Quantification of bile acids

BAs were analyzed using a Prominence^TM^ UFLC system (Shimadzu, Kyoto) coupled to an API 4000 QTRAP^TM^ mass spectrometer (AB Sciex, CA) operated in negative ionization mode based on published methods [23, 57, 58]. Chromatography was performed on a Kinetex C_18_ column (50 mm X 2.1 mm, 2.6 μm) maintained at 40°C preceded by high pressure column prefilter. The mobile phase consisted of methanol gradient delivered at a flow rate of 0.4 ml/min.

### Real-time quantitative PCR (qPCR)

Hepatic RNA isolated by TRIzol (Invitrogen, Carlsbad, CA) was reverse transcribed to generate cDNA followed by amplification using the ABI Prism 7900HT sequence detection system (Applied Biosystems, Foster City, CA). The hepatic mRNA levels were normalized to *Gpadh* mRNA levels.

### Western blot

Hepatic and ileal proteins (40 μg) were electrophoresed on SDS-polyacrylamide gels and transferred to polyvinylidene fluoride membranes. Anti-SHP, FGF15, FGF21, CYP7A1, CYP8B1, LKB1, AMPK, and β-actin (Santa Cruz, CA) antibodies were used for detection the specific proteins.

### Statistical analysis

Data are given as mean ± SD. Statistical analysis was performed using *Student's t* test or one-way analysis of variance. Significance was defined by *p* < 0.05.
